# Infants' representation of social hierarchies in absence of physical dominance

**DOI:** 10.1371/journal.pone.0245450

**Published:** 2021-02-10

**Authors:** Jesus Bas, Nuria Sebastian-Galles

**Affiliations:** Center for Brain and Cognition (CBC), Universitat Pompeu Fabra, Barcelona, Spain; Pontificia Universidad Catolica de Chile, CHILE

## Abstract

Social hierarchies are ubiquitous in all human relations since birth, but little is known about how they emerge during infancy. Previous studies have shown that infants can represent hierarchical relationships when they arise from the physical superiority of one agent over the other, but humans have the capacity to allocate social status in others through cues that not necessary entail agents’ physical formidability. Here we investigate infants’ capacity to recognize the social status of different agents when there are no observable cues of physical dominance. Our results evidence that a first presentation of the agents' social power when obtaining resources is enough to allow infants predict the outputs of their future. Nevertheless, this capacity arises later (at 18 month-olds but not at 15 month-olds) than showed in previous studies, probably due the increased complexity of the inferences needed to make the predictions.

## Introduction

The social status (or rank) refers to the position that someone has in a social hierarchy and it is based on the comparison of one agent to the others regarding a valued social dimension [1]. The recognition of social status is essential in human relations, it facilitates our interactions with others [1–4] and modulates our cognitive processes [5–7]. Most of these phenomena have been reported with adults, however humans are immersed in groups organized hierarchically since birth, for this reason in the recent years, researchers have increased their interest in understanding how humans recognize others' social status early in life. Brey and Shutts (2015) [8] found that 5 to 6 year-old children, but not younger ones, considered posture, head orientation, and eye-gaze as relevant cues to infer the social status of the individuals, Over and Carpenter (2015) [9] showed that 4 and 5-year-old children recognize others' social status on the basis of who imitated who, and Charafeddine et al. (2015) [10] showed that by 3 years of age, children infer agents’ social status on the basis of their decision power, age, and resources allocation.

Concerning the infants’ capacity to recognize hierarchical relations between different individuals, most of the studies have capitalized on the concept of social dominance. Social dominance refers to the capacity of one agent to prevail in a conflict situation [1,11,12]; that is, agents who prevail are the dominant agents (high-ranked agents). Body size as well social alliances are crucial to allocate social dominance because they increase the probability of success in agonistic fighting contexts [8,13–18]. In a seminal study, Thomsen, Frankenhuis, Ingold-Smith, & Carey (2011) [19] showed that at 10 months infants consider the size of an agent as an index of social dominance. They presented the infants with a context in which two agents, represented by geometric figures of different sizes were individually able to go from one side to the other of a platform. However, when the two agents tried to cross the platform at the same time, their paths conflicted; at that moment the agents started to bump into each other repeated times, until one of them fell down and the other could advance. Infants could see two scenarios: in one of them the bigger agent prevailed over the smaller one and in the other one the smaller agent prevailed over the bigger one. Using a violation of expectation paradigm, Thomsen et al. (2011) [19] found that infants expected the bigger agent to prevail over the smaller one. Pun et al. (2016) [20] adapted Thomsen et al. (2011)'s [19] procedure to investigate infants’ representation of social alliances in the attribution of social dominance. In their study they manipulated group size (if the conflicting agents belonged to a group of 2 or 3 members) instead of agents’ size. Their results showed that at 6 months of age, infants already expect agents belonging to larger groups to prevail in a conflict situation.

Mascaro and Csibra (2012) [21] showed infants’ capacity to represent the stability of social hierarchies across time and context. They presented 9 and 12 month-old infants with colored geometric figures representing different actors. In the familiarization phase, one of the agents moved from one side of the screen to the center and collected different items one by one. After collecting 3 items, a second same size agent appeared and every time the first agent tried to collect a new item, the second agent moved rapidly and collected the item. Therefore, that agent was considered the dominant one. In the test phase, the same two agents competed to collect a new type of item. Twelve but not 9 month-old infants expected the second (dominant) agent to prevail and to collect the new items. The authors concluded that infants considered the hierarchical relationship as stable across time. In a second experiment the authors investigated if the agents’ roles in a conflict situation were maintained and generalized through different non-related tasks. To this end, they presented 12 and 15 month-old infants with an agent who entered a walled area and remain there. Then, a second agent appeared and by pushing out the first agent “won” the position inside the walls. In the test phase, infants were presented with the same two agents competing to collect one item in an identical scenario as described for the previous experiment. The results showed that the 15 month-old infants but not the 12-month-old ones, expected the dominant agents to collect the item. These results indicated that by 15 months, infants consider the social status of an agent stable across very different contexts when physical dominance is present.

During the last years, several studies have used Mascaro and Csibra (2012)'s [21] procedure to study the scope of infants and children's representation of social hierarchies. Gazes et al. (2017) [22] explored infants' capacity to perform transitive inferences in hierarchical social relationships (see also study 4 of Mascaro and Csibra (2012) [21] and Mascaro and Csibra (2014) [23]). Enright et al. (2017) [24] explored infants' expectations of unequal resource distribution in favor of dominant agents, and Castelain et al. (2016) [25] and Bernard et al. (2016) [26] used a similar procedure to study the influence of social hierarchies on children's learning. Therefore, it is undeniable the utility of Mascaro and Csibra (2012)’s [21] procedure, which shows infants that the success of some agents prevents the success of others in conflict situations. However, a critical feature of Mascaro and Csibra (2012)’s [21] second study was the use of physical formidability to determine social status; that is, the agent who pushed stronger was the prevailing one. Although Mascaro and Csibra (2012) [21] interpreted their results as the emergence of the social status in general, the type of interactions agents were engaged in involved a relationship of physical dominance.

Humans have the capacity to establish social status through cues that do not entail the use of physical dominance, such as the knowledge, skills and success that someone shows to others and that others recognize in her [27,28]. One of the most common path to infer agents' social status is by tracking their social power, defined as their capacity to control limited resources [29,30]. Here, it is important to denote that, by definition, agents' social power is not directly correlated with agents' physical power. In the present research, we aim to investigate infants' capacity to represent the social status of individuals across different contexts, when no cues of physical dominance are presented. We hypothesize that the presentation of the agents' social power (the capacity of one agent to control a limited resource) will be enough to allow infants to make predictions about the future interactions of the agents in non-related contexts (the capacity of the same agent to control a different limited resource). Here, we will study infants' capacity to infer agents' social status by using more realistic conflict situations and social status cues. However, the lack of an observable and explicit property that justify why one agent prevails over the other (none of the agents is bigger or pushes stronger than the other) may hinder infants’ ability to represent the hierarchical relationship. Therefore, the representation of such hierarchical relationships may emerge later in infancy in comparison to studies where physical dominance is used.

We adapted the procedure of Mascaro and Csibra (2012) [21] and recorded 15- and 18-month-olds’ eye gaze while watching two physically similar agents competing to fulfill the same goal of grabbing a teddy bear. The same (high-ranked) agent always prevailed over other (low-ranked) agent after some interactions only involving eye contact [8]. Later, the same agents competed in a novel situation where the goal was to seat on an armchair. For half of the infants, the same agent (the high-ranked) kept winning (*Congruent output*) whereas for the other half, the previously lower-ranked agent won (*Incongruent output*). The selection of the first group of age was determined by the results of Mascaro and Csibra (2012) [21] who only observed generalization of social status across tasks in 15 months old. We decided to run 18 month-olds considering that from 17 months of age infants already understand that two agents could conflict to sit in a specific location [31].

## Materials and methods

**Fig 1:**
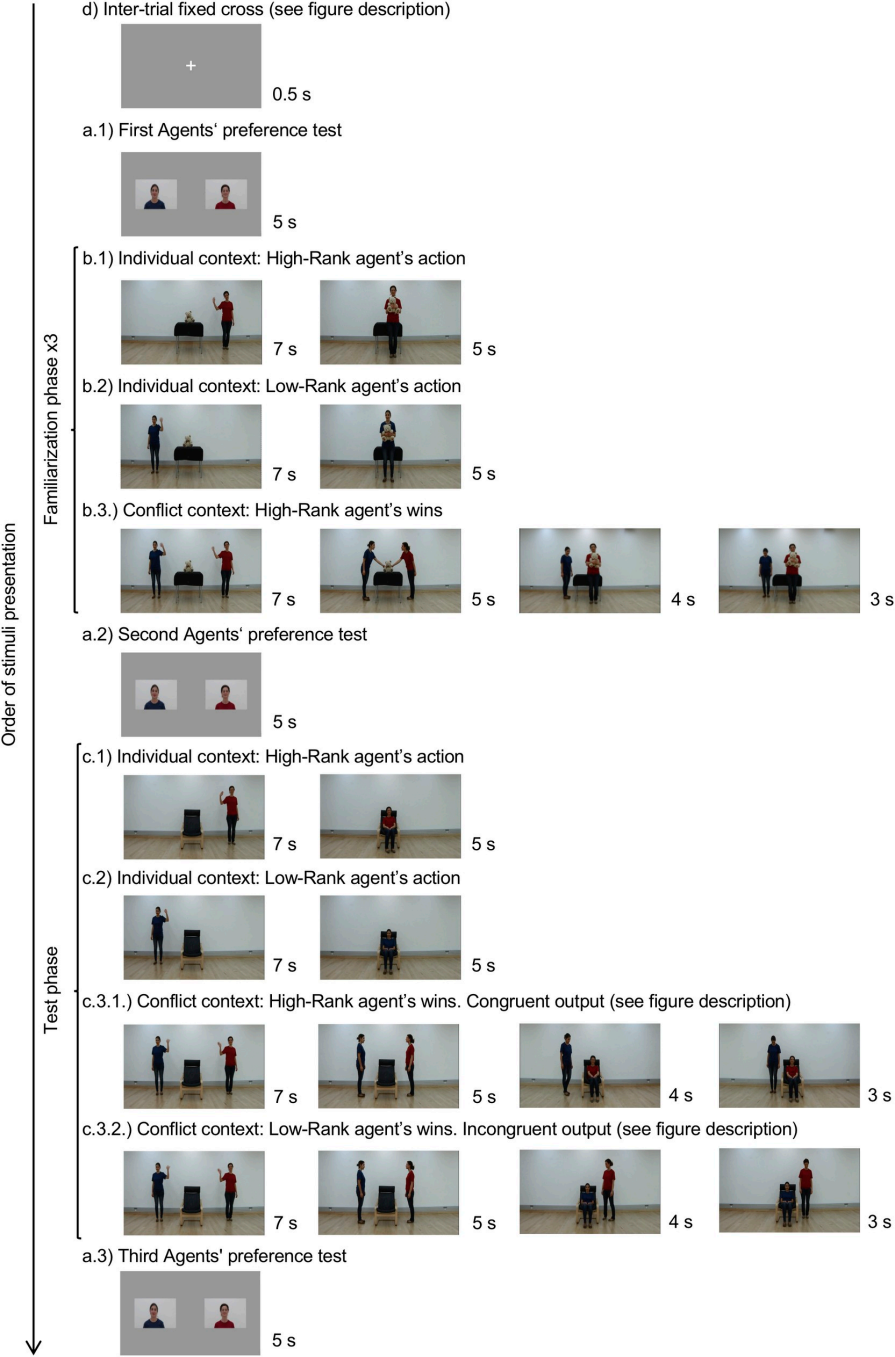
Description of the experimental procedure. The inter-trial fixed cross was presented before each stimulus appeared (d). Half of the infants saw the congruent output (c3.1), the other half the incongruent output (c3.2). See text for a description. The experiment was divided into three phases: Preference (a1, a2, a3), Familiarization (individual contexts: b1 and b2; conflict context b3) and Test (ca, c2 and c3). See the main text for a full description.

The research reported in this manuscript has been conducted in accordance with the principles expressed in the Declaration of Helsinki and approved by the local ethical committee (The Clinical Research Ethical Committee of the Parc de Salut Mar, Barcelona). All parents signed an informed consent for their infants to participate in this study. The individual pictured in Fig 1. has provided written informed consent (as outlined in PLOS consent form) to publish their image alongside the manuscript.

### Participants

The sample size was determined taking as reference previous studies [19,21] and considering that our design was between-participants. Participants were recruited by visiting maternity rooms at private hospitals, the Hospital Quirón and the Clínica Sagrada Família in Barcelona, Spain. All participants were healthy, full-term infants (> 37 Weeks of gestation).

Sixty-four infants were retained for the analysis. Thirty-two were 15 months-old, 16 (9 boys) participated in the congruent output (M = 464, SD = 14 days) and 16 (8 boys) participated in the incongruent output (M = 471, SD = 11 days). Thirty-two were 18-month-old, 16 (8 boys) participated in the congruent output (M = 550, SD = 11 days); and 16 (10 boys) participated in the incongruent output (M = 560, SD = 33 days).

Eight additional infants were tested but not included in the sample due to: technical error or experimental error (n = 1; 18-month-old), fussiness, crying or parental interference (n = 7; 4 18-month-olds). Thirty-seven additional infants were tested but not included in the sample due the quality of the eye-tracker data: less than 50% of eye-tracker data obtained during the whole experiment (n = 14; 9 18-month-olds), lack of data at one conflict-trial during the familiarization (n = 3, 1 18-month-olds), lack of data at the conflict-trial during the test phase (n = 20; 10 18-month-olds).

### Stimuli and procedure

Fig 1 illustrates the experimental procedure. The study was composed by two main phases: familiarization and test. Preference for each agent was measured at three time points during the experiment: at the beginning of the experiment, after the familiarization phase and at the end of the experiment. The social rank, as well as the side where each agent appeared on the screen, was counterbalanced across participants. Participants were randomly assigned to the different conditions.

#### Preference tests (a1, a2 and a3 in Fig 1)

A picture of each agent’s face was used to measure infants’ preference for each agent. Both agents’ pictures were presented at the same time for 5 seconds. For each infant, each agent appeared in the same side during all the experiment.

#### Familiarization phase (b1, b2 and b3 in Fig 1)

All the videos started showing the same scenario. A teddy-bear was on a table located in the middle of an empty room.

The familiarization phase was subdivided in two parts. In the first one (individual contexts, b1 and b2 in Fig 1), each agent entered the scene from one of the sides and greeted to the camera by waving her hand and smiling (7 s). Next, she looked at the teddy-bear and moved to get it. Finally, she moved forward smiling (5 s). The total video duration was 12 s. We presented first the high-ranked agent and next the low-ranked one.

In the second part (conflict context, b3 in Fig 1) both agents appeared from their corresponding sides and simultaneously greeted to the camera (7 s). Next, they looked at the teddy-bear and approached it at the same time. For the following 5 seconds, both agents performed the same sequence of actions in a synchronized way. First, they touched the teddy-bear and then they looked at each other, this action was repeated twice. Then, the high-ranked agent grabbed the teddy-bear and moved forward smiling (3 s). Then, the low-ranked agent stepped back and bended her head (4 s).

#### Test phase (c1, c2, c3.1 and c3.2 in Fig 1)

In this phase, the videos started showing an armchair in the middle of an empty room instead the teddy-bear on the table. The sequence of movements paralleled those of the familiarization phase, with two exceptions. First, the agents sat down in the armchair instead of grabbing the teddy-bear. And second, there were two possible outcomes, during the so-called critical part (framed screenshots in Fig 1), at the end of the conflict context. In the congruent output, the high-ranked agent sat down on the armchair (conflict context, c3.1 in Fig 1), and in the incongruent output, the low-ranked agent sat down on the armchair (conflict context, c3.2 in Fig 1). Half of the infants saw the congruent output, the other half the incongruent output. In this phase children only saw the sequence of the videos once.

### Apparatus

Infants were tested in the “Laboratori de Recerca en Infància”, from the Center for Brain and Cognition at Universitat Pompeu Fabra, Barcelona. Infants sat on their caregiver’s lap at approximately 65 cm from the screen in a sound-attenuated room. The session was controlled through a camera (Sony HDR-HC9E). All stimuli were presented using Matlab’s Psychtoolbox-3 software on a 23” screen and gaze was measured using a Tobii TX300 near infrared eye tracker, recording at a frequency of 300 Hz. Before each recording the eye tracker was calibrated using five-points of reference. Videos were presented in a full screen with a resolution of 1920 x 1080 pixels in a 23” screen; photos were 15 x 10 cm and they were presented at 5 cm from the center of the screen. Between each stimuli (video and images), a fixed cross (framed screenshots at d in Fig 1) at the center of the screen was presented for 0.5 seconds and the total duration of the experiment after the calibration was approximately 2 minutes and 30 seconds. Data processing were performed Matlab using. Data analyses were performed using IBM SPSS Statistics 19.

### Data analysis

**Fig 2:**
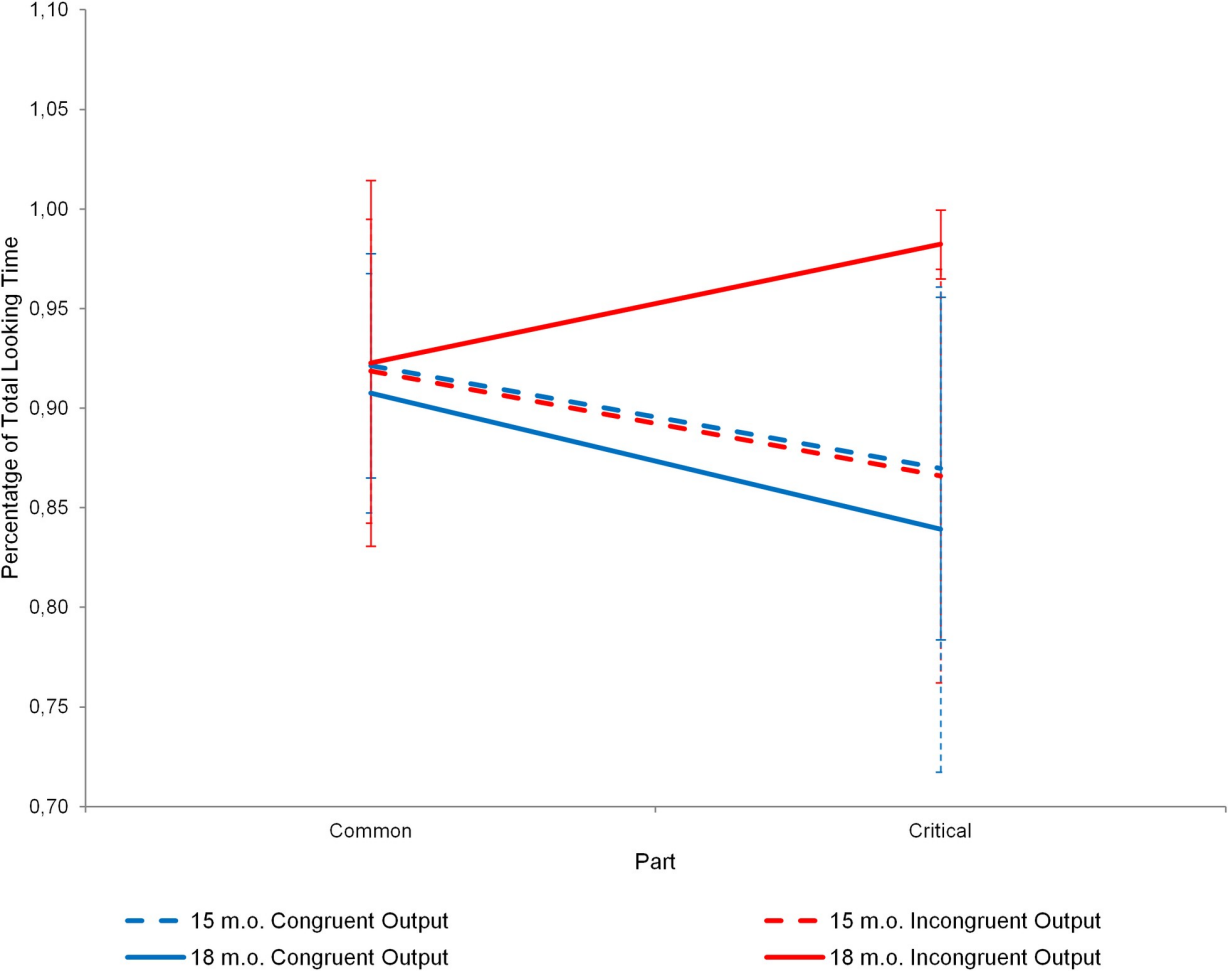
Total looking time to screen at the conflict context on the test phase across the two main parts. Error bars represent the 95% confidence interval.

The main analysis focused on the conflict context of the test phase (c3.1 and c3.2 in Fig 1). We defined two time windows according to the actions of the agents. The first time window labeled as “common” started at the beginning of the video until one of the agents took the teddy-bear or sat on the armchair (12 s). During the common window both agents performed the same actions and it served as a baseline for future comparison. The second time window called “critical” (framed screenshots at c3.1 and c3.2 in Fig 1) started when one of the agents sat on the armchair and lasted until the end of the video (7 s). We calculated the proportion of Total Looking Time to the Screen (TLTS) by dividing the time infants spent looking at the screen during each time window by the total duration thereof. This calculation was made for both congruent and incongruent outputs (see Fig 2 and Table 1 in the main text for descriptive statistics and see Table A in Supporting Information (S1) in S1 File for participants’ dataset).

**Table 1:** Mean and 95% confidence interval of the percentage of total looking time to screen at the conflict context on the test phase across the two main parts.

		Common Part Mean [CI]	Critical part Mean [CI]
**15 m.o.**	**Congruent Output**	0.921 [0.865; 0,977]	0.869 [0.783; 0.955]
	**Incongruent Output**	0.918 [0.842; 0.994]	0.865 [0.762; 0.969]
**18 m.o.**	**Congruent Output**	0.907 [0.847; 0.967]	0.839 [0.717; 0.960]
	**Incongruent Output**	0.922 [0.830; 1.014]	0.982 [0.964; 0.999]

For the preference tests (a.1, a.2 and a.3 in Fig 1) we calculated the Total Looking Time to the two Areas of Interest corresponding to the screen side occupied by the agents’ pictures (half of screen for the High-rank agent and half of the screen for the Low-rank agent). (See Table B in Supporting Information (S1) in S1 File for participants’ dataset).

## Results

A mixed repeated measure ANOVA was computed to analyze the Total Looking Time to the Screen (TLTS) based on three factors: Age (15 m.o. and 18 m.o.) and Congruency (Congruent and Incongruent) as between factors and Part (Common and Critical) as within factor (The assumption of homoscedasticity was not satisfied. Parallel analyses with normalized data [32] were performed and the pattern of results did not change. Here, we present the non-normalized analysis to ease the reading and comparison of our results with other studies.). The ANOVA showed a marginal double interaction between Congruency and Part (F (1, 60) = 3.991, p = 0.05, η^2^_*p*_ = 0.062, 1 –β = 0.931) and a significant triple interaction between Age, Congruency and Part (F (1, 60) = 4.117, p = 0.047, η^2^_*p*_ = 0.064, 1 –β = 0.938) (The relative small sample size of 16 infants may represent a limitation when interpreting three-way interactions.). Results did not reach significance in the main factors of Age, Congruence and Part, or in the interactions between Age and Congruence, and between Age and Part.

Based on our hypothesis, the triple interaction was unpacked by performing different ANOVAs for each age group separately, using Congruency and Part as factors. The interaction was only significant for 18-month-old infants (F (1, 30) = 6.478, p = 0.016, η^2^_*p*_ = 0.693, 1 –β = 1). As expected, there were no differences in the Common part between Congruent and Incongruent outputs. However, 18-month-old infants in the Incongruent Output looked longer to the screen than infants in the Congruent one during the Critical part (t (15.609) = 2.481, p = 0.025, 95% CI [-0.265, -0.020], d = 0.91, 1 –β = 0.70, Levene’s test indicated unequal variances F = 14.829, p = 0.001, so degrees of freedom were adjusted from 30 to 15.609). Similar results were obtained after deleting 3 outliers from the 18-month-old group (2 participants who looked longer than Q3 + 1.5×IQR at the Incongruent Output and 1 who looked lesser than Q1–1.5×IQR at the Congruent Output) (t (14.111) = 2.809, p = 0.0138, 95% CI [-0.197, -0.026], d = 1.08, 1 –β = 0.799, Levene’s test indicated unequal variances F = 17.378, p< 0.001, so degrees of freedom were adjusted from 27 to 14.111).

For 15-month-old infants the ANOVA showed a main effect of Part (F (1, 30) = 7.176, p = 0.012, η^2^_*p*_ = 0.736, 1 –β = 1), due the decreasing of TLTS between the Common and the Critical Part (t (31) = 2.723, p = 0.011, 95% CI [0.013, -0.091], d = 0.69, 1 –β = 0.47). There were no outliers in the 15-month-old group.

For the preference test, a mixed repeated measure ANOVA was computed to analyze Total Looking Time to the different Areas of Interest based on 3 factors: Age (15 m.o. and 18 m.o.), Area of Interest where each agent appeared (high vs low-rank agent side of the screen) and trial number (at the beginning of the experiment, after the familiarization phase and at the end of the experiment). There was no significant difference for the interaction, nor for the main effects.

## Discussion

The results of this research extend Mascaro and Csibra (2012)’s [21] results, by showing that 18-month-old, but not 15-month-old infants represent conflicts between two agents and make inferences about who is most likely to win when there are no explicit cues of physical dominance. In our experiment, 18-month-old infants and 15-month-old infants behaved in the same way when the high-raked agent won in the test phase (congruent outputs), however when the low-ranked agent won (incongruent outputs), only 18-month-old infants increased their looking times, as 15-month-old infants showed the same looking times as with the congruent outcome. These results suggest that only 18-month-old infants differentiated between the congruent and incongruent outputs and that they had expectations regarding who was most likely to prevail in the novel conflict situation. The lack of differences between the congruent and incongruent outputs in 15-month-old infants suggests that they did not surprise when the low-ranked agent prevailed. Therefore, in our study only 18-month-old infants represented the social status of the different agents as stable across the different tasks and they expected the high-ranked agent to always prevail over the low-ranked agent. These results differ from Mascaro and Csibra (2012)’s [21], as they reported that 15-month-olds were able to represent the social status of an agent as stable across different contexts. There were two main differences between our study and Mascaro and Csibra (2012)’s [21]: the type of stimuli (we used real characters and they used animated ones) and the type of information indicating agents’ social status. In our study social status was established by differences in social power to obtain resources, without any background information about why one of the agents succeeded and not the other. Although additional research should help to determine why 15 month-olds did not succeed in our study, we favor the hypothesis that the more abstract nature of agents’ hierarchical relationship during the familiarization hindered 15-month-old infants’ understanding of the situation, making it more difficult to make predictions during the test phase. We propose that in our study infants had to link agents’ social power with agents’ social status to predict the outcome in the test phase.

Social status and social power are two main concepts of hierarchical relationships that differ in their conceptualization. As said, social status is the rank that someone has in a group based on the comparison of one agent to the others and it is reflected in the way agents interact with each other [11]. Social power is the capacity of one agent to access and control specific limited resources [29]. The relationship between both concepts is asymmetrical. An individual with a high power over one resource does not have a high status if that resource is not well valued by the other agents. In addition, controlling one resource does not involve controlling others [29]. By contrast, holding a high status in a group consistently entails a high social power: the higher the social status of one individual, the more resources she will control in general. Therefore, in this conceptualization, social power is domain-specific while social status extends across different domains [30]. In our study, we familiarized infants with agents’ social power over a specific and valued resource, the teddy bear. To be able to predict the outcome in the test phase with a new type of resource, infants had to generalize agents’ social power observed in the familiarization phase to the new situation presented in the test phase (sitting in an armchair). Such generalization required the representation of agents’ social status, not directly observable in the experiment. Only by assuming that the agent's social power over one resource came from her social status, infants could predict her social power over other non-related resources. Such computation would make it difficult for the younger infants to predict who was going to prevail in the test situation.

Humans’ social hierarchies can arise by two reasons, by dominance or by prestige [27,33]. Dominant-subordinate relationships emerge when some agents impose their will over other agents, for instance, by using their physical attributes. However, prestige relationships arise from the exchange of benefits between the agents. Some agents are highly valuable for the group because of their knowledge or expertise in specific domains. For instance, in contrast to most social animals; humans learn from and are led by the most competent agents regardless of their physical features [34,35]. In return of those benefits, group members give to high competent agents a higher status by showing them respect and allowing them to get more resources (social power) [2]. Consistent with this idea, the presentation of agents’ social power without signals of physical imposition could induce the interpretation of their hierarchical relationship as part of an agreement between the agents that is, a social hierarchy based on agents' prestige. Margoni et al. (2018) [36] have recently shown that 21-month-old infants are able to recognize cues of prestige and use that information to predict agents’ actions. Although with our design we cannot determine what type of relationship infants attributed to the agents interacting, our results leave the door open to the possibility that it was agents’ social prestige. This interpretation would suggest that infants are able to interpret prestige's cues before 21 months of age; future research should focus on the relationship between prestige and social dominance and the development of their representation in infancy.

To conclude, several studies have shown that already in the first year of life, infants are able to track agents' social power and that this information allows infants to predict who is more likely to prevail in conflict scenarios. However, in many of these studies the prevailing agent showed higher physical formidability [21,23,31,37]. The relevance of the present investigation lies on the fact that the most common situation in humans' daily life is that people recognize others’ social status without witnesses the physical superiority of one agent over others. Here we present the first results showing that as early as 18 months of age infants are able to infer social status from agents’ social power. Thus, our results contribute to extend previous studies showing that during the development, humans represent competing goals and use different cues to infer agents' social status such as the size [19], social alliances [20], past social interactions in similar contexts [21], and decision power [10,26]. However, our research does not inform about the type of hierarchical relations infants represented, either based on agents’ dominance or their prestige, an important research question that future research will have to address.

## Supporting information

S1 File (DOCX)

S1 Data (XLSX)

S1 Movies (ZIP)
