# Supplementary material for: Infants' representation of social hierarchies in absence of physical dominance
Source: PLoS One. 2021 Feb 10;16(2):e0245450. doi: 10.1371/journal.pone.0245450 (PMC7875356; doi:10.1371/journal.pone.0245450)
Supplement: S1 File — (DOCX) [file pone.0245450.s001.docx]

**PLoS One Supporting Information**

Article title: Infants' representation of social hierarchies in absence of physical dominance

Authors: Jesus Bas^1^*. Nuria Sebastian-Galles ^1^

* Corresponding author; E-mai: [jesus.bas@upf.edu](mailto:jesus.bas@upf.edu)

Movies

**Movie A.** Individual context: High-Rank agent’s action

**Movie B.** Individual context: Low-Rank agent’s action

**Movie C.** Conflict context: High-Rank agent’s wins

**Movie D.** Individual context: High-Rank agent’s action

**Movie E.** Individual context: Low-Rank agent’s action

**Movie F.** Conflict context: High-Rank agent’s wins. Congruent output

**Movie G.** Conflict context: Low-Rank agent’s wins. Incongruent output

Raw data

Experimental raw data can be found at:

<http://hdl.handle.net/10230/37090>

The Raw Data file (Raw_Data_Infants.csv) is organized following the order (from left to right): Subject (subject code); Sex (1=male. 2= female); Group age (1= 15 month-old infants; 2= 18 month-old infants); Condition (description below); Video (description below); Preference repetition (description below); Validity right eye; Validity left eye; GazePoint2D.x right eye; GazePoint2D.x left eye; GazePoint2D.y right eye; GazePoint2D.y left eye (see apparatus in the main text).

*Condition* identifies the order of the stimuli presented to the participant. There were eight conditions. In conditions 1. 3. 5. 7. the outputs in the test followed the order congruent "output- incongruent output"; while in conditions 2. 4. 6. 8. the outputs in the test followed the order "incongruent output- congruent output". In conditions 1. 2. 5. 6. the high-ranked agent appeared on the left side of the screen; while in conditions 3. 4. 7. 8. the high-ranked agent appeared on the right side of the screen. The actress performing as high-ranked agent in conditions 1 to 4. performed as low-ranked agent in conditions 5 to 8.

*Video* identifies the stimulus presented on the screen (see stimuli and procedure in the main text). Videos 3. 6. 9 identify the "Individual context: High-Rank agent’s action" presented in the familiarization phase. Videos 4. 7. 9 identify the "Individual context: Low-Rank agent’s action" presented in the familiarization phase. Videos 5. 8. 11 identify the “Conflict context: High-Rank agent’s wins” presented in the familiarization phase. Video 14 identifies the "Individual context: High-Rank agent’s action" presented in the test phase. Video 15 identifies the "Individual context: Low-Rank agent’s action" presented in the familiarization phase. Video 16 identify the "Conflict context" presented in the test phase (for output order description see above *Condition*).

*Preference repetition* identifies the number of repetition of the preference test (see stimuli and procedure in the main text).

An additional data dictionary for the raw data file is provided in the supporting information excel file (File: SI_PLOSOne_Processed_Data_and_Data_Dictionaries.xlsx; Tab: “Data Dictionary Raw Data”). For further clarification contact the corresponding author.

Processed data

The Processed Data used to perform the main analyses are presented in the following tables. The same data, following an organization that facilitates its analysis, are provided in the supporting information excel file (File: SI_PLOSOne_Processed_Data_and_Data_Dictionaries.xlsx; Data dictionaries are also included).

Tables

**Table A.** Percentage of Total Looking Time to the Screen during the conflict context of the test phase. Data from Table A. are also available at the supporting information excel file (Tabs: Table A. and Data Dictionary Table A.).

|  |  | **Common Part** | **Critical part** |
| --- | --- | --- | --- |
| **15 m.o** | **Congruent Output** | 0.989 | 1.000 |
|  |  | 0.964 | 0.988 |
|  |  | 0.785 | 0.708 |
|  |  | 0.946 | 0.781 |
|  |  | 0.928 | 0.63 |
|  |  | 0.985 | 0.768 |
|  |  | 0.993 | 0.99 |
|  |  | 0.935 | 0.989 |
|  |  | 0.974 | 1.000 |
|  |  | 1.000 | 0.95 |
|  |  | 0.905 | 0.969 |
|  |  | 0.999 | 1.000 |
|  |  | 0.987 | 0.94 |
|  |  | 0.674 | 0.685 |
|  |  | 0.977 | 0.999 |
|  |  | 0.701 | 0.519 |
|  | **Incongruent Output** | 0.976 | 1.000 |
|  |  | 0.724 | 0.359 |
|  |  | 0.455 | 0.488 |
|  |  | 0.978 | 0.798 |
|  |  | 0.958 | 0.724 |
|  |  | 1.000 | 1.000 |
|  |  | 0.996 | 0.995 |
|  |  | 0.999 | 1.000 |
|  |  | 0.979 | 0.959 |
|  |  | 0.866 | 0.798 |
|  |  | 1.000 | 0.963 |
|  |  | 0.999 | 0.944 |
|  |  | 0.972 | 1.000 |
|  |  | 0.972 | 1.000 |
|  |  | 0.885 | 0.867 |
|  |  | 0.939 | 0.961 |
| **18 m.o** | **Congruent Output** | 0.592 | 0.19 |
|  |  | 0.805 | 0.697 |
|  |  | 1.000 | 0.973 |
|  |  | 0.994 | 0.999 |
|  |  | 0.893 | 0.877 |
|  |  | 0.996 | 0.999 |
|  |  | 0.804 | 0.492 |
|  |  | 0.935 | 0.996 |
|  |  | 0.981 | 0.979 |
|  |  | 0.987 | 0.88 |
|  |  | 0.989 | 0.975 |
|  |  | 0.807 | 0.918 |
|  |  | 0.889 | 0.652 |
|  |  | 0.865 | 0.822 |
|  |  | 0.984 | 0.983 |
|  |  | 0.998 | 0.994 |
|  | **Incongruent Output** | 1.000 | 0.981 |
|  |  | 0.994 | 0.984 |
|  |  | 1.000 | 0.999 |
|  |  | 0.321 | 0.895 |
|  |  | 1.000 | 1.000 |
|  |  | 0.868 | 0.999 |
|  |  | 1.000 | 1.000 |
|  |  | 0.846 | 0.909 |
|  |  | 0.998 | 1.000 |
|  |  | 0.807 | 0.977 |
|  |  | 0.983 | 1.000 |
|  |  | 0.994 | 0.976 |
|  |  | 0.974 | 0.997 |
|  |  | 1.000 | 1.000 |
|  |  | 0.989 | 1.000 |
|  |  | 0.987 | 1.000 |

**Table B.** Total Looking Time to the Areas of Interest corresponding to the screen side occupied by the agent’s pictures (L.R.: Low-Rank agent; H.R.: High-Rank agent) during the preference tests. Data from Table B. are also available in a supporting information excel file (Tabs: Table B. and Data Dictionary Table B.).

|  |  | **Repetition 1** | | **Repetition 2** | | **Repetition 3** | |
| --- | --- | --- | --- | --- | --- | --- | --- |
|  |  | **L.R.** | **H.R.** | **L.R.** | **H.R.** | **L.R.** | **H.R.** |
| **15 m.o** | **Congruent Output** | 0.244 | 0.756 | 0.426 | 0.574 | 0.429 | 0.571 |
|  |  | 0.601 | 0.399 | 0.581 | 0.419 | 0.672 | 0.328 |
|  |  |  |  | 0.592 | 0.408 | 0.473 | 0.527 |
|  |  | 1.000 | 0.000 | 0.542 | 0.458 | 0.475 | 0.525 |
|  |  | 0.468 | 0.532 | 0.000 | 1.000 | 0.563 | 0.437 |
|  |  | 0.398 | 0.602 | 0.692 | 0.308 | 0.818 | 0.182 |
|  |  | 0.297 | 0.703 | 0.251 | 0.749 | 0.401 | 0.599 |
|  |  | 0.505 | 0.495 | 0.821 | 0.179 | 0.533 | 0.467 |
|  |  | 0.632 | 0.368 | 0.461 | 0.539 | 0.660 | 0.340 |
|  |  | 0.406 | 0.594 | 0.608 | 0.392 | 0.822 | 0.178 |
|  |  | 0.436 | 0.564 | 0.555 | 0.445 | 0.556 | 0.444 |
|  |  | 0.464 | 0.536 | 0.517 | 0.483 | 0.273 | 0.727 |
|  |  | 0.560 | 0.440 | 0.687 | 0.313 | 0.564 | 0.436 |
|  |  | 0.610 | 0.390 | 0.894 | 0.106 | 0.698 | 0.307 |
|  |  | 0.501 | 0.499 | 0.653 | 0.346 | 0.644 | 0.356 |
|  |  | 0.441 | 0.559 | 0.632 | 0.368 | 0.536 | 0.464 |
|  | **Incongruent Output** | 0.436 | 0.564 | 0.426 | 0.574 | 0.515 | 0.485 |
|  |  | 0.111 | 0.889 | 0.203 | 0.797 | 0.325 | 0.675 |
|  |  | 0.250 | 1.000 | 0.479 | 0.609 | 0.481 | 0.405 |
|  |  | 0.236 | 0.764 | 0.359 | 0.641 | 0.258 | 0.742 |
|  |  | 0.653 | 0.347 | 0.621 | 0.379 | 0.701 | 0.299 |
|  |  | 0.358 | 0.642 | 0.684 | 0.316 | 0.607 | 0.393 |
|  |  | 0.527 | 0.473 | 0.673 | 0.327 | 0.475 | 0.525 |
|  |  | 0.289 | 0.711 | 0.202 | 0.798 | 0.398 | 0.602 |
|  |  | 0.460 | 0.540 | 0.630 | 0.370 | 0.567 | 0.433 |
|  |  | 0.565 | 0.435 | 0.538 | 0.462 | 0.230 | 0.770 |
|  |  | 0.540 | 0.460 | 0.575 | 0.425 | 0.543 | 0.457 |
|  |  | 0.440 | 0.560 | 0.283 | 0.717 | 0.000 | 1.000 |
|  |  | 0.518 | 0.482 | 0.623 | 0.377 | 0.565 | 0.435 |
|  |  | 0.644 | 0.356 | 0.438 | 0.562 | 0.711 | 0.289 |
|  |  |  |  | 0.809 | 0.191 | 0.504 | 0.496 |
|  |  | 0.451 | 0.549 | 0.364 | 0.636 | 0.366 | 0.634 |
| **18 m.o** | **Congruent Output** | 0.309 | 0.691 | 0.647 | 0.353 | 0.512 | 0.488 |
|  |  | 0.315 | 0.685 | 0.348 | 0.652 | 0.459 | 0.541 |
|  |  | 0.585 | 0.415 | 0.578 | 0.422 | 0.325 | 0.675 |
|  |  | 0.592 | 0.408 |  |  | 0.395 | 0.605 |
|  |  | 0.280 | 0.720 | 0.633 | 0.367 | 0.410 | 0.590 |
|  |  | 0.395 | 0.605 | 0.475 | 0.525 | 0.290 | 0.710 |
|  |  | 0.398 | 0.602 | 0.533 | 0.467 | 0.406 | 0.594 |
|  |  | 0.470 | 0.530 | 0.688 | 0.312 | 0.563 | 0.437 |
|  |  | 0.584 | 0.416 | 0.249 | 0.751 | 0.503 | 0.497 |
|  |  | 0.449 | 0.551 | 0.490 | 0.510 | 0.484 | 0.637 |
|  |  | 0.551 | 0.449 | 0.508 | 0.492 | 0.546 | 0.454 |
|  |  | 0.683 | 0.317 | 0.820 | 0.179 | 0.698 | 0.302 |
|  |  | 0.657 | 0.343 | 0.547 | 0.525 | 1.000 | 0.000 |
|  |  | 0.604 | 0.396 | 0.297 | 0.703 | 0.544 | 0.456 |
|  |  | 0.531 | 0.469 | 0.404 | 0.596 | 0.699 | 0.301 |
|  |  | 0.627 | 0.373 | 0.491 | 0.509 | 0.368 | 0.632 |
|  | **Incongruent Output** | 0.638 | 0.362 | 0.579 | 0.421 | 0.446 | 0.554 |
|  |  | 0.456 | 0.544 | 0.586 | 0.414 | 0.744 | 0.256 |
|  |  | 0.737 | 0.263 | 0.318 | 0.682 | 0.360 | 0.640 |
|  |  | 0.658 | 0.342 | 0.739 | 0.261 | 0.708 | 0.292 |
|  |  | 0.519 | 0.481 | 0.537 | 0.463 | 0.413 | 0.587 |
|  |  | 0.415 | 0.585 |  |  | 0.401 | 0.599 |
|  |  | 0.560 | 0.440 | 0.515 | 0.485 | 0.397 | 0.603 |
|  |  | 0.599 | 0.401 | 0.788 | 0.212 | 0.575 | 0.425 |
|  |  | 0.781 | 0.219 | 0.465 | 0.535 | 0.591 | 0.409 |
|  |  | 0.434 | 0.566 | 0.571 | 0.429 | 0.327 | 0.673 |
|  |  | 0.490 | 0.510 | 0.349 | 0.651 | 0.647 | 0.353 |
|  |  | 0.374 | 0.626 | 0.337 | 0.663 | 0.487 | 0.513 |
|  |  | 0.476 | 0.524 | 0.528 | 0.472 | 0.463 | 0.537 |
|  |  |  |  | 0.492 | 0.508 | 0.706 | 0.294 |
|  |  | 0.334 | 0.666 | 0.414 | 0.586 | 0.392 | 0.608 |
|  |  | 0.773 | 0.227 | 0.301 | 0.699 | 0.474 | 0.526 |
